# A Diverse Panel of Hepatitis C Virus Glycoproteins for Use in Vaccine Research Reveals Extremes of Monoclonal Antibody Neutralization Resistance

**DOI:** 10.1128/JVI.02700-15

**Published:** 2016-03-11

**Authors:** Richard A. Urbanowicz, C. Patrick McClure, Richard J. P. Brown, Theocharis Tsoleridis, Mats A. A. Persson, Thomas Krey, William L. Irving, Jonathan K. Ball, Alexander W. Tarr

**Affiliations:** a School of Life Sciences, The University of Nottingham, Nottingham University Hospitals NHS Trust, Nottingham, United Kingdom; b NIHR Nottingham Digestive Diseases Biomedical Research Unit, The University of Nottingham, Nottingham University Hospitals NHS Trust, Nottingham, United Kingdom; c Karolinska Institutet, Department of Clinical Neurosciences, Center for Molecular Medicine, Karolinska University Hospital Solna, Stockholm, Sweden; d Unité de Virologie Structurale, Département de Virologie, Institut Pasteur, Paris, France; e CNRS UMR 3569, Paris, France

## Abstract

Despite significant advances in the treatment of hepatitis C virus (HCV) infection, the need to develop preventative vaccines remains. Identification of the best vaccine candidates and evaluation of their performance in preclinical and clinical development will require appropriate neutralization assays utilizing diverse HCV isolates. We aimed to generate and characterize a panel of HCV E1E2 glycoproteins suitable for subsequent use in vaccine and therapeutic antibody testing. Full-length E1E2 clones were PCR amplified from patient-derived serum samples, cloned into an expression vector, and used to generate viral pseudoparticles (HCVpp). In addition, some of these clones were used to generate cell culture infectious (HCVcc) clones. The infectivity and neutralization sensitivity of these viruses were then determined. Bioinformatic and HCVpp infectivity screening of approximately 900 E1E2 clones resulted in the assembly of a panel of 78 functional E1E2 proteins representing distinct HCV genotypes and different stages of infection. These HCV glycoproteins differed markedly in their sensitivity to neutralizing antibodies. We used this panel to predict antibody efficacy against circulating HCV strains, highlighting the likely reason why some monoclonal antibodies failed in previous clinical trials. This study provides the first objective categorization of cross-genotype patient-derived HCV E1E2 clones according to their sensitivity to antibody neutralization. It has shown that HCV isolates have clearly distinguishable neutralization-sensitive, -resistant, or -intermediate phenotypes, which are independent of genotype. The panel provides a systematic means for characterization of the neutralizing response elicited by candidate vaccines and for defining the therapeutic potential of monoclonal antibodies.

**IMPORTANCE** Hepatitis C virus (HCV) has a global burden of more than 170 million people, many of whom cannot attain the new, expensive, direct-acting antiviral therapies. A safe and effective vaccine that generates both T cell responses and neutralizing antibodies is required to eradicate the disease. Regions within the HCV surface glycoproteins E1 and E2 are essential for virus entry and are targets for neutralizing antibodies. Screening of vaccine candidates requires suitable panels of glycoproteins that represent the breadth of neutralization resistance. Use of a standard reference panel for vaccine studies will ensure comparability of data sets, as has become routine for HIV-1. Here, we describe a large panel of patient-derived HCV glycoproteins with an assessment of their neutralization sensitivity to defined monoclonal antibodies, which has enabled us to predict their likely efficacy in the wider HCV-infected population. The panel could also be important for future selection of additional therapeutic antibodies and for vaccine design.

## INTRODUCTION

The recent development of direct-acting antiviral therapies (DAA) able to potently inhibit hepatitis C virus (HCV) replication is a major milestone toward limiting the burden of the disease, but these expensive therapies are likely to remain unattainable by the majority of the 170 million people with persistent HCV infection. Eradication of the global burden of liver disease caused by HCV infections will require the introduction of a safe, effective vaccine. While the immune correlates of vaccine-induced protection are not completely understood, generation of both effective T cell responses (1) and neutralizing antibodies (2–7) is likely to be essential. One of the major challenges in successful HCV vaccine design is the extreme genetic diversity of HCV populations (8), which results from immune-driven adaptation and escape (9, 10).

The HCV surface glycoproteins E1 and E2 are the major targets of neutralizing antibodies (reviewed in reference 11). Regions within these proteins are essential to facilitate interactions with host cell receptors during entry (12–14). This conservation and their functional importance make them highly desirable targets for therapeutic antibodies and vaccines. However, these regions are thought to be shielded by hypervariable regions, which act as immunological decoys (15, 16) and are highly glycosylated (17).

Many neutralizing monoclonal antibodies (MAbs) have been isolated from infected humans (18–22) and experimentally immunized animals (23–26). The vast majority of broadly neutralizing monoclonal antibodies target epitopes that overlap sites involved in the interaction of E2 with host CD81 (21, 27), blockading the entry cascade. Antibodies targeting other regions appear to have restricted reactivity and low neutralizing potency. An exception to this is the MAb AR4A, which recognizes a conserved neutralization epitope outside the CD81 binding region (28).

Experimental HCV glycoprotein vaccines have achieved varied levels of success (26, 29–32). Similarly, the performance of neutralizing monoclonal antibodies in clinical trials has been extremely variable (33, 34). Rational screening of lead therapeutic antibodies and vaccine candidates requires access to suitable panels of viral glycoproteins that represent the breadth of neutralization resistance. However, this has been hampered by the limited number of viruses or glycoproteins available for screening (30, 35). Provision and use of standard reference panels for vaccine and antibody studies will ensure comparability of data sets, as has become routine practice for HIV-1 (36). This will ensure that efforts can be focused on the most promising candidates and will prevent advancement of vaccines and treatments that have a high risk of failing against viruses circulating in HCV-infected populations.

Here, we describe the generation of a large panel of patient-derived HCV envelope glycoproteins from individuals at different stages of disease and infected with different genotypes, together with an assessment of their relative infectivities and neutralization sensitivity to defined monoclonal antibodies, which has enabled us to predict their likely efficacy in the wider HCV-infected population. The panel will also be important for future selection of additional therapeutic antibodies and for vaccine design (36).

## MATERIALS AND METHODS

### Glycoprotein cloning and phylogenetic analysis.

cDNA sequences encoding full-length E1E2 were amplified from RNA extracted from patient sera and cloned into the pcDNA3.1 V5his D-TOPO expression vector (Life Technologies), as previously described (37). As controls, the E1E2 genes from the widely used HCV strains H77, JFH1, and J6 were cloned into the same vector. Nucleotide sequences were determined by Sanger sequencing and aligned using Clustal W. Phylogenetic relationships were determined using maximum-likelihood analyses, as previously described (10). Bootstrap analysis with 500 replicates was performed to provide statistical support to the sequence clusters.

### Cell lines.

The human embryonic kidney 293T and Huh-7 human hepatoma cell lines were purchased from ECACC, and the Huh-7.5 cell line was obtained from Apath, LLC. All were grown in Dulbecco's modified essential medium (DMEM) (Invitrogen) supplemented with 10% fetal bovine serum (FBS) and 0.1 mM nonessential amino acids (Invitrogen).

### Antibodies.

Anti-E2 MAbs 1:7, L1, XTLAb68, and AP33 have been previously described (23, 34, 38), as has the anti-E2 nanobody (nB) D03 (26).

### HCVpp generation and infectivity and neutralization assays.

Cloned E1E2 genes were used to generate HCV pseudoparticles (HCVpp), essentially as described previously (37). Pseudoparticles generated in the absence of the E1E2 plasmid were used as a negative control. Core expression, for a subset of HCVpp production runs, was analyzed by Western blotting (35), and there were no discernible differences in expression levels (not shown). Infectivity assays were conducted as previously described (35). For neutralization assays, pseudotype virus was mixed with defined concentrations of antibody, incubated for 1 h at 37°C, and then added to Huh-7 cells. Cultures were incubated and read as for the infectivity assay. All assays were done in triplicate. The 50% inhibitory concentration (IC_50_) titer was calculated as the MAb concentration that caused a 50% reduction in relative light units (RLU) compared to the level in the virus control wells after subtraction of cell control RLU. All the data were fitted using nonlinear regression plots with no constraint on the Hill slope (GraphPad Prism version 6.05).

### HCVcc generation and neutralization assay.

Genotype 2 (Gt2) cell-culture-infectious (HCVcc) clones were generated essentially as described previously, using restriction digest cloning and PCR (39). Neutralization assays were performed as previously described (26). The percent infection was determined by comparison to the number of cells infected in the absence of inhibitors, as determined by NS5A staining with MAb 9E10 (40).

### Statistical analysis.

Viruses were compared with respect to overall neutralization sensitivity by rank ordering based on mean log_10_ IC_50_ titers across the five antibodies. A heat map describing the clustering patterns was generated using the heat map tool of the Los Alamos database (http://www.hiv.lanl.gov/content/sequence/HEATMAP/heatmap.html). The robustness of membership within a given cluster was evaluated by bootstrap resampling of the data. Frequency distribution plots fit a normal distribution curve, as shown by the D'Agostino and Pearson omnibus normality test (GraphPad Prism version 6.0). Highlighter plots were generated using the tool in the Los Alamos database (http://www.hiv.lanl.gov/content/sequence/HIGHLIGHT/highlighter_top.html).

### Nucleotide sequence accession numbers.

The E1E2 sequences described in this study have been deposited in GenBank under accession numbers KU285151 to KU285228.

## RESULTS

### Production of the HCV E1E2 glycoprotein panel.

**FIG 1:**
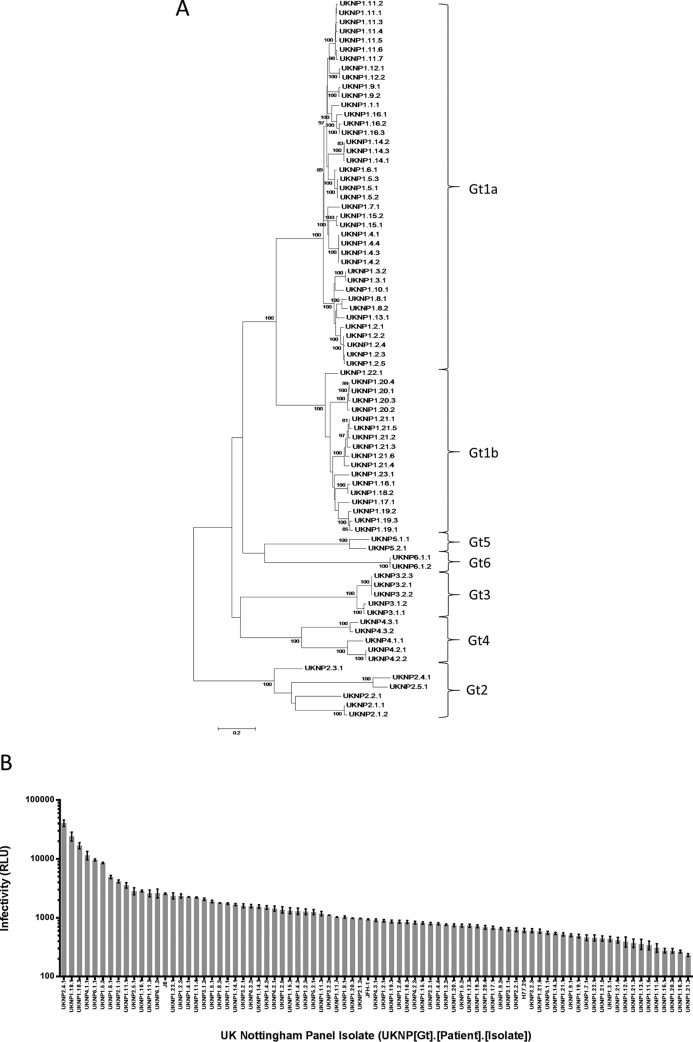
E1E2 glycoprotein clones, representing the major HCV genotypes, show varying degrees of infectivity. Shown are maximum-likelihood phylogenetic analysis (A) and relative infectivity (B) of the 81 E1E2 clones used for the antibody neutralization panel. (A) The genetic distance for each of the branch lengths shown in the phylogenetic tree is indicated by the scale bar, and the level of bootstrap support (for those branches supported by >95% of replicates) is indicated above each branch. (B) HCVpp generated without a glycoprotein envelope reproducibly gave RLU values of less than 20, and therefore, a cutoff 200 was used to determine if the clone was infectious; only those clones defined as infectious are shown. The data are mean values of triplicates ± standard deviations (SD). UKNP, United Kingdom Nottingham Panel, followed by the genotype, patient number, and isolate number.

A total of 883 E1E2 clones were generated from 3,909 patients enrolled in the Trent HCV cohort. Of these, 493 clones from 63 different subjects were sequenced and identified as containing complete E1E2 open reading frames. These were then screened for infectivity using a murine leukemia virus (MLV)-based pseudoparticle assay. One hundred eighteen clones were classified as infectious (yielding a relative luminescence value at least 10-fold greater than background). Of these, 78 clones from 36 different patients were selected for use in the neutralization panel, of which 5 (14%) were sampled during the acute early phase and 31 during chronic infection (86%) (Table 1). In addition, the standard reference strains H77, JFH-1, and J6 were included. The clones were chosen to ensure that they represented the major HCV genotypes (Fig. 1A) and a range of infectivities (Fig. 1B), although glycoproteins that conferred very low levels of infectivity were omitted from subsequent analyses because of unacceptably high intra- and interassay variability compared to an isolate with higher infectivity. Invariably, these low-infectivity clones were relatively easy to neutralize and therefore deemed less clinically relevant than neutralization-resistant strains.

**TABLE 1:** Numbers of clones analyzed from each of the six major HCV genotypes

Genotype	Total no. of clones isolated	No. of clones screened^*a*^	No. infectious clones^*a*^	No. of infectious clones in the panel^*a*^
Gt1	504	280 (32)	92 (23)	58 (23)
Gt2	85	19 (9)	8 (5)	6 (5)
Gt3	184	130 (12)	7 (3)	5 (2)
Gt4	35	19 (3)	6 (3)	5 (3)
Gt5	51	36 (6)	3 (2)	2 (2)
Gt6	24	9 (1)	2 (1)	2 (1)
Total	883	493 (63)	118 (37)	78 (36)

a Total number (number of unique subjects).

### Estimated inhibitory concentrations of antibody are consistent between experimental runs.

**FIG 2:**
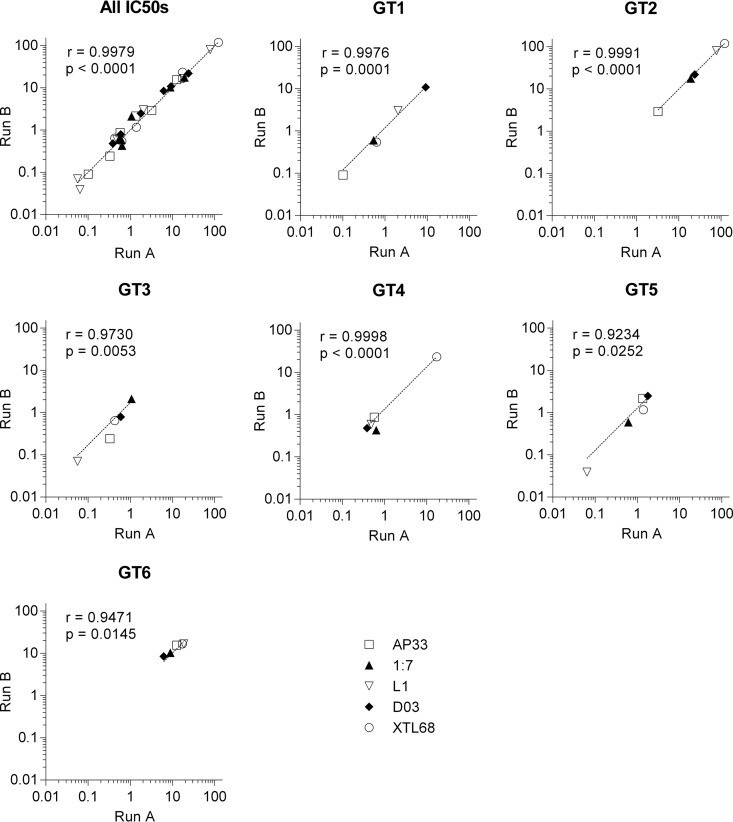
The estimated inhibitory concentrations of antibodies are consistent between different neutralization assay runs. IC_50_s for MAb AP33, MAb 1:7, MAb L1, nAb D03, and XTL68, estimated from two independent neutralization assays using HCVpp supplemented with E1E2 proteins representing the six major genotypes, were compared to assess the impact of interassay variability. The IC_50_ titer for each repeat assay was plotted for the entire data set (All IC_50_s) or for each of the 6 HCVpp. The solid lines show the best fit of the experimental data. Pearson correlation coefficient *R* values are shown, along with the associated *P* values.

Due to the large number of clones included in the neutralization assays and the inherent variability of RLU values that could be observed between experimental runs, we compared IC_50_s between two independent neutralization assays to allow us to evaluate interassay reproducibility. HCVpp supplemented with six clones previously shown to exhibit different neutralization resistance phenotypes were tested in two separate neutralization assays using the same CD81 binding site MAbs that were to be used for the subsequent analyses of the full panel. There was good correlation between the two different runs for all of the antibodies tested (Fig. 2), irrespective of the genotype or the magnitude of the IC_50_. These results show that our experimental approach was subject to low run-to-run variability.

### Patient-derived glycoprotein clones of the same subtype can differ markedly in their sensitivity to neutralization.

**FIG 3:**
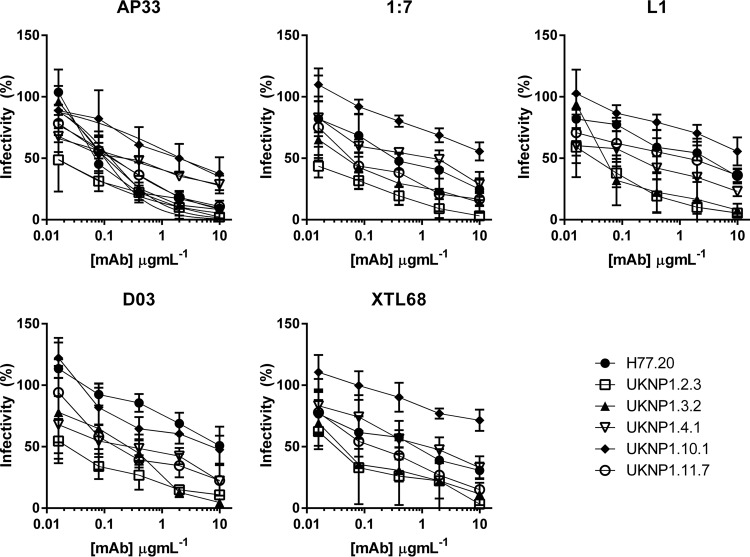
Genotype 1 clones show both resistant and sensitive phenotypes. HCVpp supplemented with genotype 1 E1E2 proteins H77.20, UKNP1.2.3, UKNP1.3.2, UKNP1.4.1, UKNP1.10.1, and UKNP1.11.7 were neutralized by increasing concentrations of MAb AP33, MAb 1:7, MAb L1, nB D03, and MAb XTL68, and the resulting infectivity was plotted against the antibody concentration.

From the larger panel of 81 infectious clones, a subset of 5 genotype 1 clones, together with the reference molecular clone H77, were tested against four MAbs (AP33 [41], 1:7 [38], L1 [38], and XTL68 [34]) and one nanobody (nB D03 [26]), all of which target discrete epitopes located in the CD81 binding site. The IC_50_s are presented in Table 2, and the corresponding neutralization curves are presented in Fig. 3. Sensitivity to neutralization varied across the six genotype 1 isolates, with some being highly sensitive to the antibodies (e.g., UKNP1.2.3) while others were highly resistant (e.g., UKNP1.10.1). These data strongly support the view that sensitivity to a neutralizing antibody is determined at the isolate rather than the genotype level.

**TABLE 2:** IC_50_s of neutralization curves in Fig. 3

Clone	IC_50_ (μg ml^−1^)^*a*^				
	MAb AP33	MAb 1:7	MAb L1	nB D03	MAb XTL68
H77.20	0.1012	0.5388	2.031	9.167	0.6316
UKNP1.2.3	0.01467	0.009883	0.03123	0.02079	0.03315
UKNP1.3.2	0.1294	0.0553	0.06801	0.1913	0.05287
UKNP1.4.1	0.2245	0.8219	0.1371	0.2545	1.27
UKNP1.10.1	2.118	11.88	15.47	5.091	44.02
UKNP1.11.7	0.1408	0.1022	0.99	0.3288	0.1833

a The IC_50_ for each genotype 1 HCVpp/antibody curve was calculated using nonlinear regression plots with no constraint on the Hill slope (GraphPad Prism version 6.05).

### Extensive variability in neutralization sensitivity of patient-derived envelope glycoproteins.

**FIG 4:**
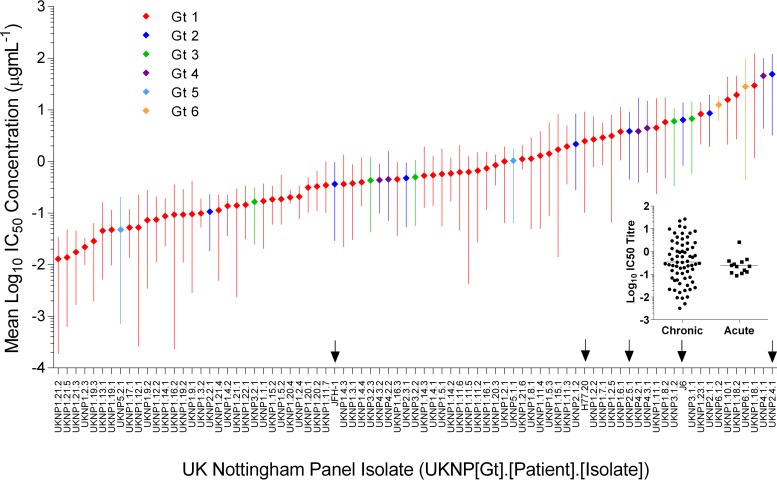
Pseudotype viruses incorporating different E1E2 clones derived from the major HCV genotypes display diverse susceptibilities to antibody neutralization. Eighty-one distinct patient-derived and reference E1E2 clones were assessed for neutralization sensitivities using five monoclonal antibodies. The mean log_10_ IC_50_ neutralization value, and the range, for each HCVpp supplemented with E1E2 derived from genotypes 1, 2, 3, 4, 5, and 6 were plotted in rank order from lowest (most sensitive) to highest (least sensitive). The arrows indicate the clones that were used in the subsequent HCVcc analysis. The inset graph shows the mean neutralization IC_50_s for E1E2 genes sampled from either acute or chronic HCV infection. Both groups possessed similar neutralization resistance phenotypes, as shown by a one-way analysis of variance.

**FIG 5:**
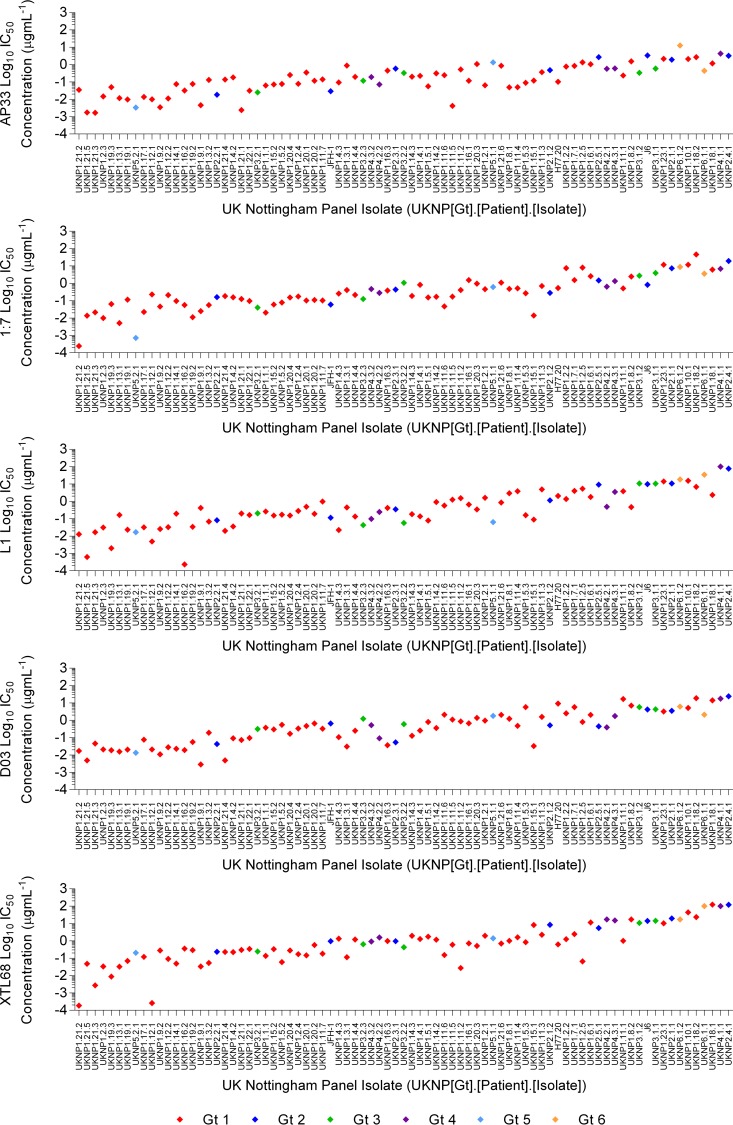
Pseudotype viruses incorporating different E1E2 clones derived from the major HCV genotypes display diverse susceptibilities to antibody neutralization. Eighty-one distinct patient-derived and reference E1E2 clones derived from genotypes 1, 2, 3, 4, 5, and 6 were assessed for their neutralization sensitivity to five monoclonal antibodies. The log_10_ IC_50_ for MAbs AP33, 1:7, L1, and XTL68 and nB D03 are plotted according to the E1E2 clone's rank order presented in Fig. 4.

Neutralization assays were performed to determine the sensitivity of each of the 81 clones in the larger glycoprotein panel to neutralization by each of the five antibodies, and the means of the resulting IC_50_s for E1E2 HCVpp clones were calculated and plotted, together with their ranges, in mean rank order (Fig. 4). These mean IC_50_s constituted a spectrum. The inset shows that when the isolates are split into chronic and acute early phase samples, there is no significant difference in the log_10_ IC_50_s between the groups. The most easily neutralized E1E2 clone (UKNP1.21.2) had a mean IC_50_ of 0.013 μg ml^−1^, whereas the most resistant clone (UKNP2.4.1) had a mean IC_50_ of 49 μg ml^−1^. When the neutralization sensitivities of the clones were analyzed according to individual antibodies (Fig. 5), their relative positions in the neutralization spectrum were on the whole maintained, indicating an inherent sensitivity to neutralization by CD81 binding site-targeting antibodies. However, a few clones had greater sensitivity to some antibodies than their relative positions in the plot of mean IC_50_s would suggest. For example, UKNP1.16.2 was very susceptible to neutralization by MAb L1 (Fig. 5), while UKNP1.12.1 was very susceptible to neutralization by MAb XTL68 (Fig. 5). No neutralization was observed when antibodies were tested against a negative control (vesicular stomatitis virus G [VSV-G]) or when pseudoparticles were assayed using anti-tetanus toxin MAb (Wyeth) (data not shown). The mean IC_50_s (±standard deviations) for MAbs AP33, 1:7, L1, D03, and XTL68 were 0.6872 (±1.587), 2.105 (±5.909), 4.581 (±14.59), 2.289 (±4.600), and 8.887 (±24.69) μg ml^−1^, respectively.

### The neutralization phenotypes of patient E1E2 proteins are consistent in both HCVpp and HCVcc models of infection.

**FIG 6:**
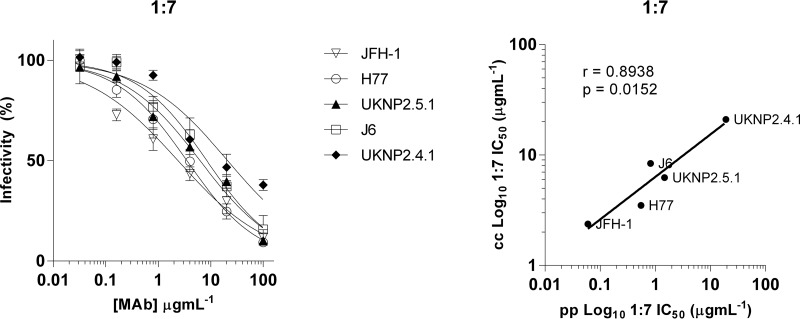
A subset of E1E2 clones transferred to a chimeric HCVcc infection model has neutralization sensitivity concordant with that in the HCVpp model. (Left) Two E1E2 clones showing high resistance in the HCVpp panel were transferred into the J6/JFH-1 backbone and neutralized, along with J6, JFH-1, and H77, with increasing concentrations of MAb 1:7. The error bars indicate SD. (Right) The IC_50_s for both the HCVcc and HCVpp showed significant correlation (*r* = 0.8938; *P* = 0.0152).

To determine if the same pattern of neutralization sensitivity also applied to the cell culture model, two Gt2 E1E2 clones from the panel were transferred into the J6/JFH-1 cell-culture-infectious HCV backbone (Fig. 4, arrows). These clones and three reference strains, JFH-1, J6/JFH-1, and H77/JFH-1, represented a breadth of neutralization sensitivities, as predicted from the HCVpp panel, and were tested against MAb 1:7 (Fig. 6, left, and Table 3). Even with this small subset, there was a significant correlation (*r* = 0.8938; *P* = 0.0152) between the neutralization data obtained in the HCVpp and HCVcc systems (Fig. 6, right).

**TABLE 3:** Comparison of neutralization IC_50_s of patient-isolated clones in both the HCVpp and HCVcc infection models

HCVcc clone	Neutralization rank^*a*^	IC_50_ (μg ml^−1^)	
		HCVpp 1:7	HCVcc 1:7
JFH-1	32	0.06	2.37
H77.20	60	0.55	3.51
UKNP2.5.1	65	1.47	6.25
J6	71	0.82	8.41
UKNP2.4.1	81	19.11	21.05

a Position in the ranking of the mean IC_50_ HCVpp neutralization values presented in Fig. 4.

### Increased infectivity conferred by E1E2 clones is associated with increased neutralization resistance.

**FIG 7:**
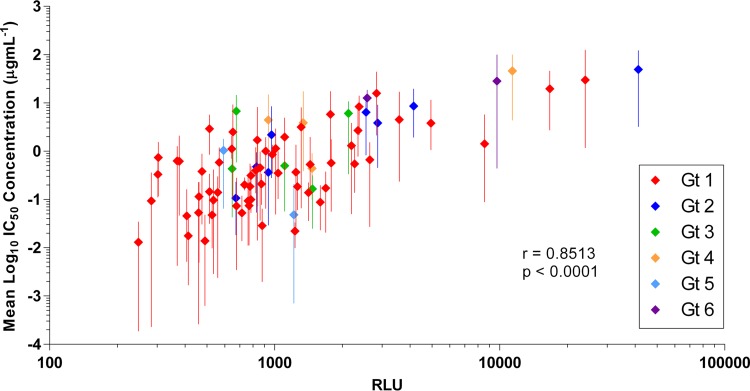
HCVpp infectivity conferred by E1E2 clones is correlated with its resistance to antibody neutralization. The mean uninhibited infectivity (RLU value), and the range, for each of the 81 E1E2 clones supplementing HCVpp was plotted against the mean log_10_ IC_50_ neutralization titer. *r* = 0.8513; *P* < 0.0001.

To investigate the relationship between infectivity and neutralization sensitivity, the mean log_10_ IC_50_s were plotted against the relative infectivity (as defined by the RLU value) of each of the E1E2 clones (Fig. 7). There was a direct correlation between increased neutralization resistance, as indicated by increasing IC_50_s, and increased infectivity, and this correlation was statistically significant (*r* = 0.8513; *P* < 0.0001). This trend was also maintained when the analysis was performed using individual MAb IC_50_ data (not shown). E1E2 clone UKNP2.4.1 had the highest level of infectivity, and it was also the most resistant to antibody neutralization.

### The glycoprotein panel can be used to predict the likely efficacy of monoclonal antibodies against circulating HCV strains.

**FIG 8:**
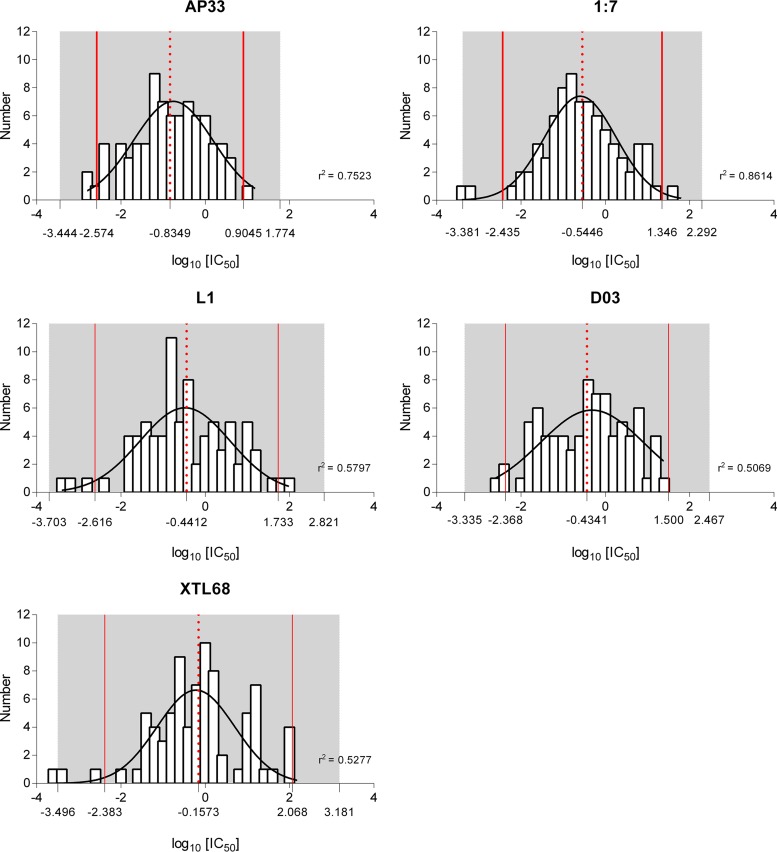
Normally distributed IC_50_s for each antibody. Shown are frequency distribution plots of log_10_ IC_50_s, and the derived Gaussian curves, from all 81 clones for MAbs AP33, 1:7, L1, and XTL68 and nB D03. The mean log_10_ IC_50_ for each antibody is represented by a red dashed line, the mean ± 2 standard deviations by solid red lines, and the mean ± 3 standard deviations by gray shading. Each plot fit a normal distribution curve, as shown by the D'Agostino and Pearson omnibus normality test.

An important aim of this study was to determine whether the glycoprotein panel could be used to inform future vaccine and therapeutic antibody efficacy by predicting likely neutralizing potencies against a wider set of patient-derived isolates. Plots of log_10_ IC_50_s for each antibody showed a normal distribution (Fig. 8), meaning that each antibody plot could be used to predict an antibody concentration that would exceed the IC_50_ for 95% (equal to the mean of the neutralization distribution plus 2 standard deviations) or 99% (mean plus 3 standard deviations) of HCV isolates (Table 4). For MAb AP33, a concentration of 8 μg ml^−1^ would be sufficient to meet the IC_50_s of 95% of circulating HCV strains, but this would need to be increased to 59 μg ml^−1^ in order to effectively neutralize 99% of circulating isolates. In comparison, concentrations of XTL68 of 117 μg ml^−1^ and 1.51 mg ml^−1^ would be needed to provide the same level of neutralization for 95% and 99% of circulating HCV isolates, respectively.

**TABLE 4:** IC_50_s from frequency distribution plots in Fig. 5

Antibody	IC_50_ (μg ml^−1^)^*a*^	
	95% HCV isolates	99% HCV isolates
AP33	8	59
1:7	22	196
L1	54	662
D03	32	293
XTL68	117	1,517

a IC_50_s were estimated from the frequency distribution plots based on the mean values plus 2 standard deviations (95%) or mean values plus 3 standard deviations (99%).

### Patient-isolated E1E2 can be categorized into distinct neutralization phenotype clusters.

**FIG 9:**
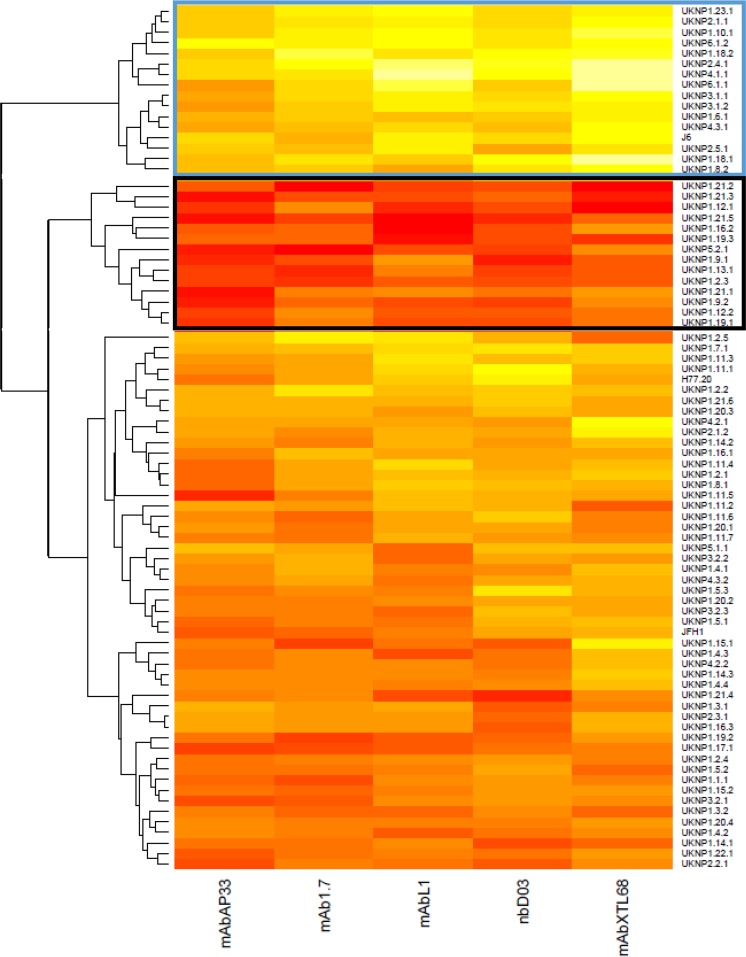
In hierarchical cluster analysis, the E1E2 clones can be grouped according to their sensitivity to monoclonal antibody neutralization. HCV E1E2 pseudoviruses (*n* = 81) were assessed for neutralization sensitivities using five MAbs targeting discrete epitopes of the CD81 binding site. Individual clones are listed on the right, and antibodies are indicated at the base of the heat map. The magnitude of neutralization (log_10_ IC_50_ titer) is denoted by the color, where higher values of neutralization are represented by lighter colors (e.g., light yellows) and lower values are represented by more saturated, dark colors (e.g., dark red). Boxes are drawn around viral isolates that were resistant (blue) and sensitive (black) to neutralization that grouped with >90% probability according to bootstrap resampling (10,000 replicates) of the data set.

**FIG 10:**
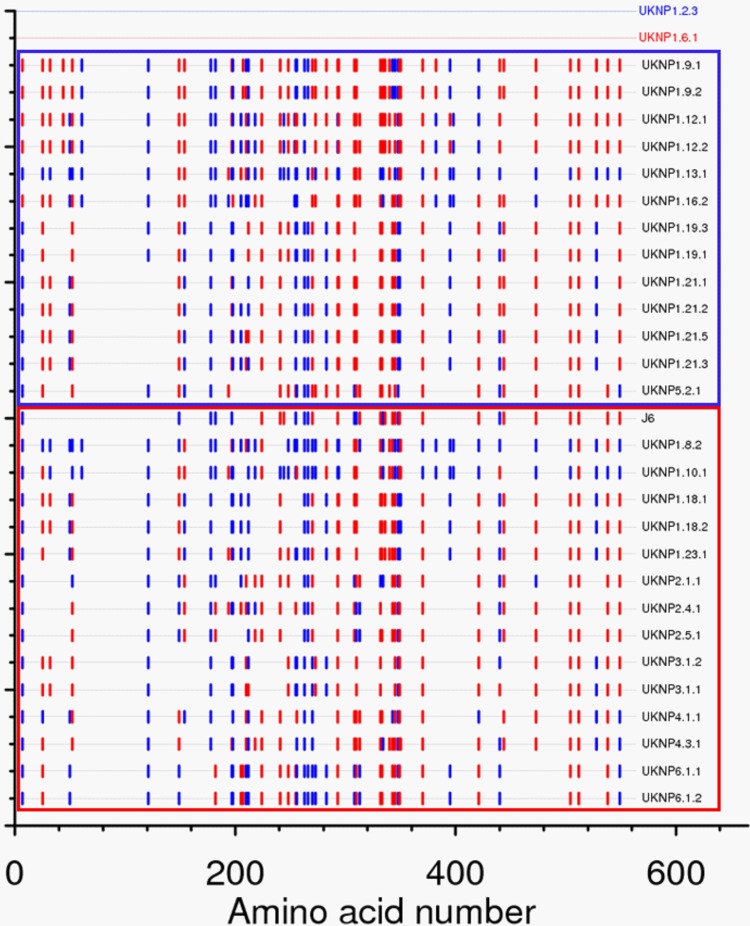
Highlighter analysis of matches between clones categorized as neutralization resistant or neutralization sensitive. The two clusters of clones identified in Fig. 7 were compared using the highlighter tool, with strains UKNP1.2.3 and UKNP1.6.1 as references for neutralization-sensitive and -resistant phenotypes, respectively. Sensitive isolates are boxed in blue, while resistant isolates are boxed in red. Matches to UKNP1.2.3 are indicated in blue, and matches to UKNP1.6.1 are indicated in red. No consistent pattern of matches within the two groups was observed.

In order to categorize the E1E2 panel into groups based on similar neutralization phenotypes, we next performed hierarchical cluster analysis of the IC_50_ titers for each isolate/MAb. The E1E2 clones segregated into three distinct groups (Fig. 9). One group consisted of 16 E1E2 clones that were highly resistant to neutralization (blue box), one consisted of 14 highly sensitive E1E2 isolates (black box), and the third group consisted of isolates with an intermediate phenotype. Each cluster contained a mixture of different genotypes, once again highlighting the fact that the genotype has little, if any, bearing on sensitivity to neutralization. The mean (±standard deviation) of IC_50_s observed for the resistant cluster was 15.9 (±14.74) μg ml^−1^ compared to 0.05468 (±0.03714) μg ml^−1^ for the group of sensitive viruses. This highlighted the extreme differences in inherent neutralization sensitivity between patient-derived isolates. Analyzed separately, the mean IC_50_s for each antibody also showed large differences between the two groups (Table 5). To establish if there were any common amino acids in the E1E2 genes that conferred the resistant or sensitive phenotype, highlighter analysis was performed to identify matches with a selected resistant (UKNP1.6.1) and sensitive (UKNP1.2.3) clone. However, no common amino acid predicted either of the two phenotypes (Fig. 10), suggesting that neutralization resistance can occur through multiple mechanisms.

**TABLE 5:** Mean IC_50_s for the resistant and sensitive neutralization clusters for all antibodies and individual antibodies^*a*^

Cluster	Mean IC_50_ (μg ml^−1^)					
	Combined	AP33	1:7	L1	D03	XTL68
Sensitive	0.05468 (0.03714)	0.0136 (0.01475)	0.06553 (0.07598)	0.0675 (0.1175)	0.02213 (0.0177)	0.1046 (0.1257)
Resistant	15.9 (14.74)	2.541 (2.903)	8.545 (11.11)	20.39 (28.23)	7.501 (7.053)	40.52 (43.51)

a Numbers in parentheses are standard deviations of the means.

## DISCUSSION

Future HCV vaccines or antibody-based therapies will need to be effective against the majority, if not all, of the viruses present in infected individuals. This requires good preclinical screening of interventions to identify the best leads, which in turn relies upon the availability of reference panels of patient-derived HCV isolates. While some groups have reported successful culture of patient-derived HCV, these methods generally exhibit poor replication efficiency and therefore do not represent a tractable means of predicting neutralization sensitivity *in vivo*; instead, surrogate models, such as the HCVpp system or chimeric HCVcc, have proven to be more robust tools (reviewed in reference 42). Previous studies on antibody neutralization have used the cell line Huh7.5 (40) or primary hepatocytes (43); we chose the former, as that cell line is more amenable and the HCV entry process is comparable to that observed in primary hepatocytes (44–46).

We had previously shown that patient-derived E1E2 clones were differentially susceptible to neutralization by human polyclonal sera, and this appeared to be unrelated to the genotype (35). Here, we extend those preliminary studies by generating a significantly larger panel of E1E2 HCVpp clones, drawn from different genotypes that are representative of individuals at different stages of infection. This marks a major advancement in the field, where existing knowledge of protective antibody determinants has been derived from much smaller or genotype-restricted HCVpp or HCVcc panels (28, 39, 47, 48). Importantly, our data highlight the fact that intra- or intergenotypic differences do not noticeably impact sensitivity to neutralization (at least not with respect to CD81 binding site-targeting MAbs) and that this property is determined at the isolate level. In our initial screening, we identified a number of clones that were not functional in the HCVpp system. This has been observed previously (12), and the reasons for this lack of functionality remain unclear.

One of the most striking observations was the extreme variability in neutralization sensitivity that patient-derived E1E2 clones demonstrated. While many E1E2 clones showed similar degrees of sensitivity to CD81 binding site-targeting MAbs, there were a number of isolates that were especially sensitive or resistant. Comparisons between resistant and sensitive strains failed to identify common sequence substitutions or motifs giving rise to this difference. Identifying mechanisms of resistance will be important in understanding neutralizing antibody responses, although it is likely that sequence differences underlying these different neutralization phenotypes will be highly isolate specific.

While our panel contained predominantly subtype 1a and 1b viruses, there was a sufficiently large number of clones representing the other major genotypes to be confident that the panel could provide a robust prediction of the neutralizing potency of individual MAbs against HCV strains present in infected individuals. For example, the panel has enabled us to estimate that 8 μg ml^−1^ of MAb AP33, which equals the mean IC_50_ plus 2 standard deviations, will provide at least 50% neutralization for ∼95% of patient-derived isolates, whereas for XTL68, this would increase to 117 μg ml^−1^. Importantly, this type of screening strategy provides insight into the likely potency of any given MAb when used against any patient-derived isolate. Given the observed correlation between *in vitro* and *in vivo* anti-HCV activities of MAbs (49, 50), this approach could be used to provide a quick indication of an antibody's potential clinical value. In this respect, it is important to note that in a previous clinical trial, MAb XTL68 had only modest effect when administered to HCV-infected individuals (34). It is also important to note that other factors, such as the potential for MAb-driven escape (51), are also important parameters in determining the likely clinical value of any particular antibody or antibody combination. Finally, resistance to MAb neutralization was correlated with the degree of infectivity, as determined by the HCVpp assay. Such a relationship between increased antibody neutralization resistance and improved HCVpp infectivity has been reported previously in analyses of polyclonal sera obtained from a small cohort of liver transplant recipients (43).

Having established that isolates showed a wide range of neutralization sensitivities, we performed cluster analysis to define clusters of MAb sensitivity. The bulk of isolates showed variable patterns of neutralization—easily neutralized by some but not all MAbs tested. However, there were two distinct clusters that represented the two extremes, one highly sensitive and one highly resistant group. Both clusters contained viruses drawn from highly divergent genotypes. The mean IC_50_, for all five antibodies, of the resistant group was 290 times greater than that observed for the sensitive group. This was also true for the antibodies individually. The resistant viruses pose a major challenge for vaccine development and cannot be ignored; otherwise, deployment of a less than optimal vaccine would result in the selection and eventual dissemination of a virus population refractive to neutralization, as has been observed in the most promising HIV-1 vaccine trial to date (52).

Importantly, two strains (H77c and JFH-1) that have been used extensively to test the potency of candidate vaccine-induced sera fall outside the neutralization-resistant cluster. In contrast, the J6 reference strain is a more relevant reference strain to include in this type of analysis, which probably explains the variable neutralization observed for E1E2-elicited immune sera (29). This is consistent with the antibody neutralization resistance of this clone that was evident, but not commented upon, in previous studies (29, 53).

HCVpp represents a rapid, amenable way to test antibody neutralization against a large number of different isolates. Importantly, we have shown, at least for a subset of isolates, that the HCVpp-derived neutralization data accords with those obtained using chimeric HCVcc. While significant advances have been made in generating more diverse panels of infectious virus, it is still a very laborious process, so the range of isolates available is still very limited (28, 39). Although the HCVpp system provides greater flexibility with respect to the number of HCV isolates that can be tested easily, our data show that while there was good correlation between the two systems, HCVpp were more easily neutralized than the corresponding HCVcc chimeras. Therefore, while HCVpp is an easy system for rapid screening of MAbs and sera, the findings would need to be validated in the HCVcc system. Also, HCVcc chimeras utilizing more clinically relevant isolates are required for *in vivo* studies. Therefore, creation of chimeras of the clones representing resistant-cluster isolates is under way.

Our study focused on an initial characterization of the panel, using MAbs that targeted the CD81 binding site. These MAbs represent the majority of murine and human cross-neutralizing anti-HCV MAbs. Differences in sensitivity to antibodies and polyclonal sera targeting other neutralizing determinants will be important to assess in the future. However, it is important to reiterate that in our previous analysis of a much smaller panel using polyclonal sera, a similar spectrum of neutralization was observed (35).

In conclusion, this study provides the first objective categorization of cross-genotype patient-derived HCV E1E2 clones according to their sensitivity to antibody neutralization and showed that individual HCV isolates can be categorized as having a clearly distinguishable neutralization-sensitive, -resistant, or -intermediate phenotype. We were able to use the panel to predict the likely efficacies of a number of CD81 binding site MAbs against circulating HCV strains. This reference panel will be indispensable for future studies of the therapeutic potential of existing and newly discovered MAbs, used alone or in combination, as well as the potency of immune-generated sera.

## References

[1] Thimme R, Bukh J, Spangenberg HC, Wieland S, Pemberton J, Steiger C, Govindarajan S, Purcell RH, Chisari FV 2002 Viral and immunological determinants of hepatitis C virus clearance, persistence, and disease. Proc Natl Acad Sci U S A 99:15661–15668. doi:10.1073/pnas.202608299. 12441397 PMC137773

[2] Netski DM, Mosbruger T, Depla E, Maertens G, Ray SC, Hamilton RG, Roundtree S, Thomas DL, McKeating J, Cox A 2005 Humoral immune response in acute hepatitis C virus infection. Clin Infect Dis 41:667–675. doi:10.1086/432478. 16080089

[3] Osburn WO, Snider AE, Wells BL, Latanich R, Bailey JR, Thomas DL, Cox AL, Ray SC 2014 Clearance of hepatitis C infection is associated with the early appearance of broad neutralizing antibody responses. Hepatology 59:2140–2151. doi:10.1002/hep.27013. 24425349 PMC4043926

[4] Osburn WO, Fisher BE, Dowd KA, Urban G, Liu L, Ray SC, Thomas DL, Cox AL 2010 Spontaneous control of primary hepatitis C virus infection and immunity against persistent reinfection. Gastroenterology 138:315–324. doi:10.1053/j.gastro.2009.09.017. 19782080 PMC2889495

[5] Raghuraman S, Park H, Osburn WO, Winkelstein E, Edlin BR, Rehermann B 2012 Spontaneous clearance of chronic hepatitis C virus infection is associated with appearance of neutralizing antibodies and reversal of T-cell exhaustion. J Infect Dis 205:763–771. doi:10.1093/infdis/jir835. 22293431 PMC3274373

[6] Pestka JM, Zeisel MB, Blaser E, Schurmann P, Bartosch B, Cosset FL, Patel AH, Meisel H, Baumert J, Viazov S, Rispeter K, Blum HE, Roggendorf M, Baumert TF 2007 Rapid induction of virus-neutralizing antibodies and viral clearance in a single-source outbreak of hepatitis C. Proc Natl Acad Sci U S A 104:6025–6030. doi:10.1073/pnas.0607026104. 17392433 PMC1851610

[7] Youn JW, Park SH, Lavillette D, Cosset FL, Yang SH, Lee CG, Jin HT, Kim CM, Shata MT, Lee DH, Pfahler W, Prince AM, Sung YC 2005 Sustained E2 antibody response correlates with reduced peak viremia after hepatitis C virus infection in the chimpanzee. Hepatology 42:1429–1436. doi:10.1002/hep.20934. 16317673

[8] Ruhl M, Knuschke T, Schewior K, Glavinic L, Neumann-Haefelin C, Chang DI, Klein M, Heinemann FM, Tenckhoff H, Wiese M, Horn PA, Viazov S, Spengler U, Roggendorf M, Scherbaum N, Nattermann J, Hoffmann D, Timm J, East German HCV Study Group 2011 CD8+ T-cell response promotes evolution of hepatitis C virus nonstructural proteins. Gastroenterology 140:2064–2073. doi:10.1053/j.gastro.2011.02.060. 21376049

[9] Bull RA, Luciani F, McElroy K, Gaudieri S, Pham ST, Chopra A, Cameron B, Maher L, Dore GJ, White PA, Lloyd AR 2011 Sequential bottlenecks drive viral evolution in early acute hepatitis C virus infection. PLoS Pathog 7:e1002243. doi:10.1371/journal.ppat.1002243. 21912520 PMC3164670

[10] Brown RJ, Hudson N, Wilson G, Rehman SU, Jabbari S, Hu K, Tarr AW, Borrow P, Joyce M, Lewis J, Zhu LF, Law M, Kneteman N, Tyrrell DL, McKeating JA, Ball JK 2012 Hepatitis C virus envelope glycoprotein fitness defines virus population composition following transmission to a new host. J Virol 86:11956–11966. doi:10.1128/JVI.01079-12. 22855498 PMC3486514

[11] Ball JK, Tarr AW, McKeating JA 2014 The past, present and future of neutralizing antibodies for hepatitis C virus. Antiviral Res 105:100–111. doi:10.1016/j.antiviral.2014.02.013. 24583033 PMC4034163

[12] Lavillette D, Tarr AW, Voisset C, Donot P, Bartosch B, Bain C, Patel AH, Dubuisson J, Ball JK, Cosset FL 2005 Characterization of host-range and cell entry properties of the major genotypes and subtypes of hepatitis C virus. Hepatology 41:265–274. doi:10.1002/hep.20542. 15660396

[13] Owsianka AM, Timms JM, Tarr AW, Brown RJ, Hickling TP, Szwejk A, Bienkowska-Szewczyk K, Thomson BJ, Patel AH, Ball JK 2006 Identification of conserved residues in the E2 envelope glycoprotein of the hepatitis C virus that are critical for CD81 binding. J Virol 80:8695–8704. doi:10.1128/JVI.00271-06. 16912317 PMC1563869

[14] Drummer HE, Boo I, Maerz AL, Poumbourios P 2006 A conserved Gly436-Trp-Leu-Ala-Gly-Leu-Phe-Tyr motif in hepatitis C virus glycoprotein E2 is a determinant of CD81 binding and viral entry. J Virol 80:7844–7853. doi:10.1128/JVI.00029-06. 16873241 PMC1563787

[15] Bankwitz D, Steinmann E, Bitzegeio J, Ciesek S, Friesland M, Herrmann E, Zeisel MB, Baumert TF, Keck ZY, Foung SK, Pecheur EI, Pietschmann T 2010 Hepatitis C virus hypervariable region 1 modulates receptor interactions, conceals the CD81 binding site, and protects conserved neutralizing epitopes. J Virol 84:5751–5763. doi:10.1128/JVI.02200-09. 20357091 PMC2876602

[16] Mondelli MU, Cerino A, Segagni L, Meola A, Cividini A, Silini E, Nicosia A 2001 Hypervariable region 1 of hepatitis C virus: immunological decoy or biologically relevant domain? Antiviral Res 52:153–159. doi:10.1016/S0166-3542(01)00180-2. 11672825

[17] Helle F, Duverlie G, Dubuisson J 2011 The hepatitis C virus glycan shield and evasion of the humoral immune response. Viruses 3:1909–1932. doi:10.3390/v3101909. 22069522 PMC3205388

[18] Broering TJ, Garrity KA, Boatright NK, Sloan SE, Sandor F, Thomas WD Jr, Szabo G, Finberg RW, Ambrosino DM, Babcock GJ 2009 Identification and characterization of broadly neutralizing human monoclonal antibodies directed against the E2 envelope glycoprotein of hepatitis C virus. J Virol 83:12473–12482. doi:10.1128/JVI.01138-09. 19759151 PMC2786766

[19] Schofield DJ, Bartosch B, Shimizu YK, Allander T, Alter HJ, Emerson SU, Cosset FL, Purcell RH 2005 Human monoclonal antibodies that react with the E2 glycoprotein of hepatitis C virus and possess neutralizing activity. Hepatology 42:1055–1062. doi:10.1002/hep.20906. 16250048

[20] Meunier JC, Russell RS, Goossens V, Priem S, Walter H, Depla E, Union A, Faulk KN, Bukh J, Emerson SU, Purcell RH 2008 Isolation and characterization of broadly neutralizing human monoclonal antibodies to the e1 glycoprotein of hepatitis C virus. J Virol 82:966–973. doi:10.1128/JVI.01872-07. 17977972 PMC2224608

[21] Perotti M, Mancini N, Diotti RA, Tarr AW, Ball JK, Owsianka A, Adair R, Patel AH, Clementi M, Burioni R 2008 Identification of a broadly cross-reacting and neutralizing human monoclonal antibody directed against the hepatitis C virus E2 protein. J Virol 82:1047–1052. doi:10.1128/JVI.01986-07. 17989176 PMC2224572

[22] Keck ZY, Op De Beeck A, Hadlock KG, Xia J, Li TK, Dubuisson J, Foung SK 2004 Hepatitis C virus E2 has three immunogenic domains containing conformational epitopes with distinct properties and biological functions. J Virol 78:9224–9232. doi:10.1128/JVI.78.17.9224-9232.2004. 15308717 PMC506923

[23] Owsianka A, Tarr AW, Juttla VS, Lavillette D, Bartosch B, Cosset FL, Ball JK, Patel AH 2005 Monoclonal antibody AP33 defines a broadly neutralizing epitope on the hepatitis C virus E2 envelope glycoprotein. J Virol 79:11095–11104. doi:10.1128/JVI.79.17.11095-11104.2005. 16103160 PMC1193588

[24] Vieyres G, Dubuisson J, Patel AH 2011 Characterization of antibody-mediated neutralization directed against the hypervariable region 1 of hepatitis C virus E2 glycoprotein. J Gen Virol 92:494–506. doi:10.1099/vir.0.028092-0. 21084495 PMC3081231

[25] Hsu M, Zhang J, Flint M, Logvinoff C, Cheng-Mayer C, Rice CM, McKeating JA 2003 Hepatitis C virus glycoproteins mediate pH-dependent cell entry of pseudotyped retroviral particles. Proc Natl Acad Sci USA 100:7271–7276. doi:10.1073/pnas.0832180100. 12761383 PMC165865

[26] Tarr AW, Lafaye P, Meredith L, Damier-Piolle L, Urbanowicz RA, Meola A, Jestin JL, Brown RJ, McKeating JA, Rey FA, Ball JK, Krey T 2013 An alpaca nanobody inhibits hepatitis C virus entry and cell-to-cell transmission. Hepatology 58:932–939. doi:10.1002/hep.26430. 23553604

[27] Owsianka AM, Tarr AW, Keck ZY, Li TK, Witteveldt J, Adair R, Foung SK, Ball JK, Patel AH 2008 Broadly neutralizing human monoclonal antibodies to the hepatitis C virus E2 glycoprotein. J Gen Virol 89:653–659. doi:10.1099/vir.0.83386-0. 18272755 PMC2885755

[28] Giang E, Dorner M, Prentoe JC, Dreux M, Evans MJ, Bukh J, Rice CM, Ploss A, Burton DR, Law M 2012 Human broadly neutralizing antibodies to the envelope glycoprotein complex of hepatitis C virus. Proc Natl Acad Sci U S A 109:6205–6210. doi:10.1073/pnas.1114927109. 22492964 PMC3341081

[29] Wong JA, Bhat R, Hockman D, Logan M, Chen C, Levin A, Frey SE, Belshe RB, Tyrrell DL, Law JL, Houghton M 2014 Recombinant hepatitis C virus envelope glycoprotein vaccine elicits antibodies targeting multiple epitopes on the envelope glycoproteins associated with broad cross-neutralization. J Virol 88:14278–14288. doi:10.1128/JVI.01911-14. 25275133 PMC4249152

[30] Chmielewska AM, Naddeo M, Capone S, Ammendola V, Hu K, Meredith L, Verhoye L, Rychlowska M, Rappuoli R, Ulmer JB, Colloca S, Nicosia A, Cortese R, Leroux-Roels G, Balfe P, Bienkowska-Szewczyk K, Meuleman P, McKeating JA, Folgori A 2014 Combined adenovirus vector and hepatitis C virus envelope protein prime-boost regimen elicits T cell and neutralizing antibody immune responses. J Virol 88:5502–5510. doi:10.1128/JVI.03574-13. 24599994 PMC4019094

[31] Stamataki Z, Coates S, Evans MJ, Wininger M, Crawford K, Dong C, Fong YL, Chien D, Abrignani S, Balfe P, Rice CM, McKeating JA, Houghton M 2007 Hepatitis C virus envelope glycoprotein immunization of rodents elicits cross-reactive neutralizing antibodies. Vaccine 25:7773–7784. doi:10.1016/j.vaccine.2007.08.053. 17919789

[32] Stamataki Z, Coates S, Abrignani S, Houghton M, McKeating JA 2011 Immunization of human volunteers with hepatitis C virus envelope glycoproteins elicits antibodies that cross-neutralize heterologous virus strains. J Infect Dis 204:811–813. doi:10.1093/infdis/jir399. 21788452 PMC3203385

[33] Chung RT, Gordon FD, Curry MP, Schiano TD, Emre S, Corey K, Markmann JF, Hertl M, Pomposelli JJ, Pomfret EA, Florman S, Schilsky M, Broering TJ, Finberg RW, Szabo G, Zamore PD, Khettry U, Babcock GJ, Ambrosino DM, Leav B, Leney M, Smith HL, Molrine DC 2013 Human monoclonal antibody MBL-HCV1 delays HCV viral rebound following liver transplantation: a randomized controlled study. Am J Transplant 13:1047–1054. doi:10.1111/ajt.12083. 23356386 PMC3618536

[34] Schiano TD, Charlton M, Younossi Z, Galun E, Pruett T, Tur-Kaspa R, Eren R, Dagan S, Graham N, Williams PV, Andrews J 2006 Monoclonal antibody HCV-AbXTL68 in patients undergoing liver transplantation for HCV: results of a phase 2 randomized study. Liver Transplantation 12:1381–1389. doi:10.1002/lt.20876. 16933235

[35] Tarr AW, Urbanowicz RA, Hamed MR, Albecka A, McClure CP, Brown RJ, Irving WL, Dubuisson J, Ball JK 2011 Hepatitis C patient-derived glycoproteins exhibit marked differences in susceptibility to serum neutralizing antibodies: genetic subtype defines antigenic but not neutralization serotype. J Virol 85:4246–4257. doi:10.1128/JVI.01332-10. 21325403 PMC3126256

[36] Seaman MS, Janes H, Hawkins N, Grandpre LE, Devoy C, Giri A, Coffey RT, Harris L, Wood B, Daniels MG, Bhattacharya T, Lapedes A, Polonis VR, McCutchan FE, Gilbert PB, Self SG, Korber BT, Montefiori DC, Mascola JR 2010 Tiered categorization of a diverse panel of HIV-1 Env pseudoviruses for assessment of neutralizing antibodies. J Virol 84:1439–1452. doi:10.1128/JVI.02108-09. 19939925 PMC2812321

[37] Tarr A, Owsianka A, Szwejk A, Ball J, Patel A 2007 Cloning, expression, and functional analysis of patient-derived hepatitis C virus glycoproteins. Methods Mol Biol 379:177–197. doi:10.1007/978-1-59745-393-6_13. 17502679

[38] Johansson DX, Voisset C, Tarr AW, Aung M, Ball JK, Dubuisson J, Persson MA 2007 Human combinatorial libraries yield rare antibodies that broadly neutralize hepatitis C virus. Proc Natl Acad Sci U S A 104:16269–16274. doi:10.1073/pnas.0705522104. 17911260 PMC2042196

[39] Gottwein JM, Scheel TKH, Jensen TB, Lademann JB, Prentoe JC, Knudsen ML, Hoegh AM, Bukh J 2009 Development and characterization of hepatitis C virus genotype 1-7 cell culture systems: role of CD81 and scavenger receptor class B type I and effect of antiviral drugs. Hepatology 49:364–377. doi:10.1002/hep.22673. 19148942

[40] Lindenbach BD, Evans MJ, Syder AJ, Wolk B, Tellinghuisen TL, Liu CC, Maruyama T, Hynes RO, Burton DR, McKeating JA, Rice CM 2005 Complete replication of hepatitis C virus in cell culture. Science 309:623–626. doi:10.1126/science.1114016. 15947137

[41] Tarr AW, Owsianka AM, Timms JM, McClure CP, Brown RJ, Hickling TP, Pietschmann T, Bartenschlager R, Patel AH, Ball JK 2006 Characterization of the hepatitis C virus E2 epitope defined by the broadly neutralizing monoclonal antibody AP33. Hepatology 43:592–601. doi:10.1002/hep.21088. 16496330

[42] Lohmann V, Bartenschlager R 2014 On the history of hepatitis C virus cell culture systems. J Med Chem 57:1627–1642. doi:10.1021/jm401401n. 24164647

[43] Fafi-Kremer S, Fofana I, Soulier E, Carolla P, Meuleman P, Leroux-Roels G, Patel AH, Cosset F-L, Pessaux P, Doffoël M, Wolf P, Stoll-Keller F, Baumert TF 2010 Viral entry and escape from antibody-mediated neutralization influence hepatitis C virus reinfection in liver transplantation. J Exp Med 207:2019–2031. doi:10.1084/jem.20090766. 20713596 PMC2931157

[44] Wilson GK, Farquhar MJ, Meredith L, Dhawan A, Mitry R, Balfe P, McKeating JA 2015 Permissivity of primary hepatocytes and hepatoma cell lines to support hepatitis C virus infection. J Gen Virol 96:1369–1373. doi:10.1099/vir.0.000085. 25667327 PMC4635487

[45] Régeard M, Trotard M, Lepère C, Gripon P, Le Seyec J 2008 Entry of pseudotyped hepatitis C virus into primary human hepatocytes depends on the scavenger class B type I receptor. J Viral Hepat 15:865–870. doi:10.1111/j.1365-2893.2008.01048.x. 19087225

[46] Codran A, Royer C, Jaeck D, Bastien-Valle M, Baumert TF, Kieny MP, Pereira CA, Martin J-P 2006 Entry of hepatitis C virus pseudotypes into primary human hepatocytes by clathrin-dependent endocytosis. J Gen Virol 87:2583–2593. doi:10.1099/vir.0.81710-0. 16894197

[47] Pedersen J, Carlsen THR, Prentoe J, Ramirez S, Jensen TB, Forns X, Alter H, Foung SKH, Law M, Gottwein J, Weis N, Bukh J 2013 Neutralization resistance of hepatitis C virus can be overcome by recombinant human monoclonal antibodies. Hepatology 58:1587–1597. doi:10.1002/hep.26524. 23729237 PMC4415732

[48] Bailey JR, Wasilewski LN, Snider AE, El-Diwany R, Osburn WO, Keck Z, Foung SKH, Ray SC 2015 Naturally selected hepatitis C virus polymorphisms confer broad neutralizing antibody resistance. J Clin Invest 125:437–447. doi:10.1172/JCI78794. 25500884 PMC4382262

[49] Meuleman P, Catanese MT, Verhoye L, Desombere I, Farhoudi A, Jones CT, Sheahan T, Grzyb K, Cortese R, Rice CM, Leroux-Roels G, Nicosia A 2012 A human monoclonal antibody targeting scavenger receptor class B type I precludes hepatitis C virus infection and viral spread in vitro and in vivo. Hepatology 55:364–372. doi:10.1002/hep.24692. 21953761 PMC3262867

[50] Mailly L, Xiao F, Lupberger J, Wilson GK, Aubert P, Duong FH, Calabrese D, Leboeuf C, Fofana I, Thumann C, Bandiera S, Lutgehetmann M, Volz T, Davis C, Harris HJ, Mee CJ, Girardi E, Chane-Woon-Ming B, Ericsson M, Fletcher N, Bartenschlager R, Pessaux P, Vercauteren K, Meuleman P, Villa P, Kaderali L, Pfeffer S, Heim MH, Neunlist M, Zeisel MB, Dandri M, McKeating JA, Robinet E, Baumert TF 2015 Clearance of persistent hepatitis C virus infection in humanized mice using a claudin-1-targeting monoclonal antibody. Nat Biotechnol 33:549–554. doi:10.1038/nbt.3179. 25798937 PMC4430301

[51] Pantua H, Diao J, Ultsch M, Hazen M, Mathieu M, McCutcheon K, Takeda K, Date S, Cheung TK, Phung Q, Hass P, Arnott D, Hongo J-A, Matthews DJ, Brown A, Patel AH, Kelley RF, Eigenbrot C, Kapadia SB 2013 Glycan shifting on hepatitis C virus (HCV) E2 glycoprotein is a mechanism for escape from broadly neutralizing antibodies. J Mol Biol 425:1899–1914. doi:10.1016/j.jmb.2013.02.025. 23458406

[52] Rolland M, Edlefsen PT, Larsen BB, Tovanabutra S, Sanders-Buell E, Hertz T, deCamp AC, Carrico C, Menis S, Magaret CA, Ahmed H, Juraska M, Chen L, Konopa P, Nariya S, Stoddard JN, Wong K, Zhao H, Deng W, Maust BS, Bose M, Howell S, Bates A, Lazzaro M, O'Sullivan A, Lei E, Bradfield A, Ibitamuno G, Assawadarachai V, O'Connell RJ, deSouza MS, Nitayaphan S, Rerks-Ngarm S, Robb ML, McLellan JS, Georgiev I, Kwong PD, Carlson JM, Michael NL, Schief WR, Gilbert PB, Mullins JI, Kim JH 2012 Increased HIV-1 vaccine efficacy against viruses with genetic signatures in Env V2. Nature 490:417–420. doi:10.1038/nature11519. 22960785 PMC3551291

[53] Sabo MC, Luca VC, Prentoe J, Hopcraft SE, Blight KJ, Yi M, Lemon SM, Ball JK, Bukh J, Evans MJ, Fremont DH, Diamond MS 2011 Neutralizing monoclonal antibodies against hepatitis C virus E2 protein bind discontinuous epitopes and inhibit infection at a postattachment step. J Virol 85:7005–7019. doi:10.1128/JVI.00586-11. 21543495 PMC3126585

